# Progress Toward Poliomyelitis Eradication — Pakistan, January 2021–July 2022

**DOI:** 10.15585/mmwr.mm7142a1

**Published:** 2022-10-21

**Authors:** Chukwuma Mbaeyi, Shahzad Baig, Muhammad Rana Safdar, Zainul Khan, Hamish Young, Jaume Jorba, Zubair M. Wadood, Hamid Jafari, Muhammad Masroor Alam, Richard Franka

**Affiliations:** ^1^Global Immunization Division, Center for Global Health, CDC; ^2^National Emergency Operation Center, Islamabad, Pakistan; ^3^World Health Organization, Islamabad, Pakistan; ^4^UNICEF, Islamabad, Pakistan; ^5^Division of Viral Diseases, National Center for Immunization and Respiratory Diseases, CDC; ^6^Polio Eradication Department, World Health Organization, Geneva, Switzerland; ^7^World Health Organization, Amman, Jordan.

After reporting a single wild poliovirus (WPV) type 1 (WPV1) case in 2021, Pakistan reported 14 cases during April 1–July 31, 2022. Pakistan and Afghanistan are the only countries where endemic WPV transmission has never been interrupted (*1*). In its current 5-year strategic plan, the Global Polio Eradication Initiative (GPEI) has set a goal of interrupting all WPV1 transmission by the end of 2023 (*1*–*3*). The reemergence of WPV cases in Pakistan after 14 months with no case detection has uncovered transmission in southern Khyber Pakhtunkhwa province, the most historically challenging area. This report describes Pakistan’s progress toward polio eradication during January 2021–July 2022 and updates previous reports (*4*,*5*). As of August 20, 2022, all but one of the 14 WPV1 cases in Pakistan during 2022 have been reported from North Waziristan district in Khyber Pakhtunkhwa. In underimmunized populations, excretion of vaccine virus can, during a period of 12–18 months, lead to reversion to neurovirulence, resulting in circulating vaccine-derived polioviruses (cVDPVs), which can cause paralysis and outbreaks. An outbreak of cVDPV type 2 (cVDPV2), which began in Pakistan in 2019, has been successfully contained; the last case occurred in April 2021 (*1*,*6*). Despite program improvements, 400,000–500,000 children continue to be missed during nationwide polio supplementary immunization activities (SIAs),* and recent isolation of poliovirus from sewage samples collected in other provinces suggests wider WPV1 circulation during the ongoing high transmission season. Although vaccination efforts have been recently complicated by months of flooding during the summer of 2022, to successfully interrupt WPV1 transmission in the core reservoirs in southern Khyber Pakhtunkhwa and reach the GPEI goal, emphasis should be placed on further improving microplanning and supervision of SIAs and on systematic tracking and vaccination of persistently missed children in these reservoir areas of Pakistan.

## Immunization Activities

**Essential (routine) immunization.** The World Health Organization (WHO) and UNICEF estimated Pakistan’s 2021 national polio vaccination coverage (3 doses of oral poliovirus vaccine [OPV] and 1 dose of inactivated poliovirus vaccine by age 12 months) at 83% (*7*). A 2021 survey sponsored by WHO and Gavi, the Vaccine Alliance,^†^ indicated that the proportion of children aged 12–23 months who had received 3 OPV doses, by province, ranged from 45.1% in Balochistan to 94.9% in Punjab. None of the districts in the provinces of Balochistan, Khyber Pakhtunkhwa, and Sindh achieved ≥80% coverage. By comparison, 31 of 36 (86%) districts in Punjab province achieved ≥80% 3-dose OPV coverage.

**Supplementary immunization activities.** After the declaration of eradication of WPV type 2 in 2015,^§^ Pakistan joined other countries in GPEI in implementing a synchronized withdrawal of trivalent OPV (tOPV; containing Sabin-strain types 1, 2, and 3) in 2016 as part of containment efforts for all type 2 polioviruses (*8*). However, with the emergence of cVDPV2 in Pakistan in 2019, GPEI authorized the use of tOPV along with the recommended monovalent Sabin-strain OPV type 2 (mOPV2) for outbreak response vaccination activities. During 2021, 4 national immunization days (NIDs) and 2 subnational immunization days (SNIDs) directed at children aged <5 years were conducted using bivalent OPV (bOPV; containing Sabin-strain types 1 and 3) and, in areas with cVDPV2 transmission, either mOPV2 or tOPV. The November 2021 NIDs were combined with a measles-rubella vaccination campaign that reached 90 million persons aged 9 months–15 years with measles-rubella vaccine in addition to doses of bOPV administered to 41 million children aged <5 years.

Two NIDs (in March and May) and 2 SNIDs (in January and June) targeting children aged <5 years have been conducted to date in 2022 using bOPV. SNIDs took place in designated, high-risk districts for poliovirus transmission and other priority areas for the polio program mostly in the provinces of Khyber Pakhtunkhwa, Balochistan, Sindh, and Punjab. Limited SIAs were conducted in response to identification of WPV1 cases and environmental isolates in March, April, and June. An NID was conducted in August; another is planned for November, and an SNID is planned for October 2022.

During SIAs conducted in 25 very high-risk districts,^¶^ including approximately 10–12 million children aged <5 years, the number of children missed because the child was absent from the household declined 9%, from 184,597 in September 2021 to 167,934 in May 2022, and the number of refusals among eligible children decreased 23%, from 66,875 to 51,577. Collectively, among 43 million children targeted during each NID, 400,000–500,000 (0.9%–1.2%) children were repeatedly being missed. Lot quality assurance sampling (LQAS)** survey results have also indicated performance gaps in districts identified to be at highest risk for poliovirus transmission. Only 67%–80% of these districts reached the 90% LQAS threshold for SIAs conducted during September 2021–May 2022.

## Poliovirus Surveillance

**Acute flaccid paralysis surveillance**. Detection of two or more cases of nonpolio acute flaccid paralysis (AFP) per 100,000 children and adolescents aged <15 years per year is an indicator of adequately sensitive polio surveillance.^††^ Pakistan reported a national nonpolio AFP rate of 13 per 100,000 children and adolescents in 2021; provincial rates ranged from 9.5 to 18.8. As of July 27, 2022, the annualized 2022 nonpolio AFP rate was 14.2; stool adequacy^§§^ rates, a measure of completeness of case investigation, was ≥80% nationally and in each province during 2021 and 2022.

**Environmental surveillance**. Laboratory testing of sewage samples routinely collected at designated sites supplements AFP surveillance in facilitating timely detection of circulating polioviruses. Pakistan has 77 environmental surveillance sampling sites. During 2021, 65 (8%) of 833 sewage samples tested positive for WPV1 compared with 407 (52%) of 786 samples tested in 2020. In 2022, to date, 13 (2%) of 748 samples have tested positive for WPV1, including eight from Khyber Pakhtunkhwa province, four from Punjab province, and one from Islamabad. The earliest isolates detected in samples collected from environmental surveillance sites in Bannu district (Khyber Pakhtunkwa) in April 2022 were orphan viruses (i.e., ≥1.5% divergent from their closest genetic match), indicating gaps in AFP surveillance sensitivity; subsequent isolates were genetically linked to WPV1 cases detected in North Waziristan.

**FIGURE 1:**
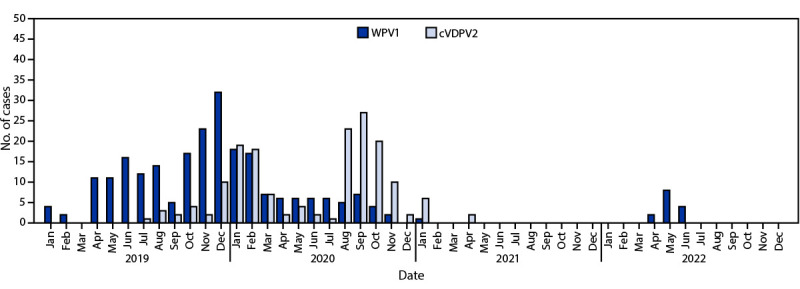
Wild poliovirus type 1 and circulating vaccine-derived poliovirus type 2 cases, by month — Pakistan, January 2019–July 2022 **Abbreviations:** cVDPV2 = circulating vaccine-derived poliovirus type 2; WPV1 = wild poliovirus type 1.

**FIGURE 2:**
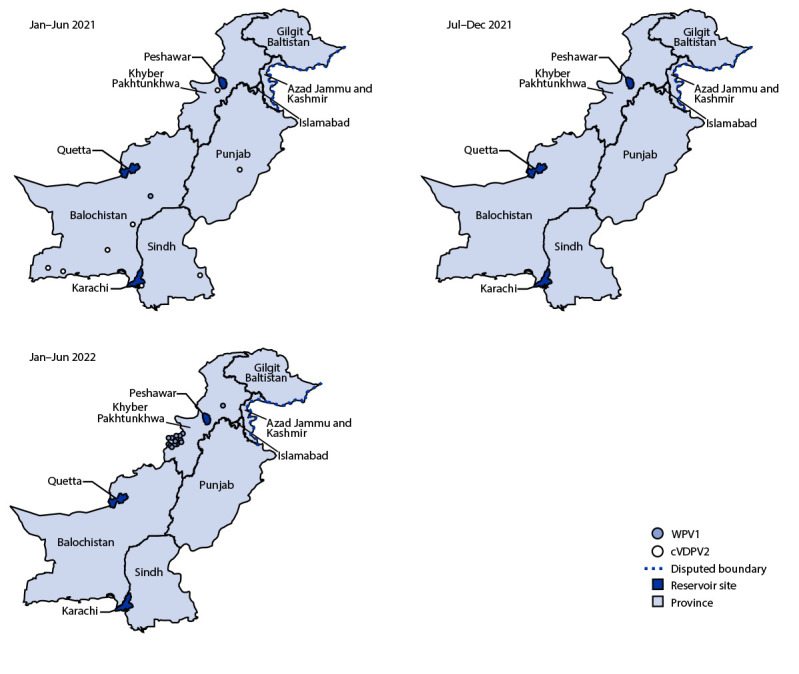
Location of cases of wild poliovirus type 1 and circulating vaccine-derived poliovirus type 2, by province and period — Pakistan, January 2021–June 2022 **Abbreviations:** cVDPV2 = circulating vaccine-derived poliovirus type 2; WPV1 = wild poliovirus type 1.

**Epidemiology of poliomyelitis cases.** During 2021, a single Pakistan WPV1 case was reported in Killa Abdullah, Balochistan, compared with 84 cases reported from several provinces in 2020 and 147 cases reported during 2019 (Figure 1). As of August 20, 2022, 14 WPV1 cases had been reported from two districts in Khyber Pakhtunkhwa in 2022, including North Waziristan (13) and Lakki Marwat (one) (Figure 2).

Of the 15 WPV1 cases reported during January 2021–July 2022, patients’ ages ranged from 7 to 28 months (median = 15 months); 87% had never received OPV through essential immunization (zero-dose children), and 13% had received 1–3 OPV doses through essential immunization. Genetic analysis of the viruses identified in the WPV1 cases indicated that all belong to a single genetic cluster (groups of polioviruses sharing ≥95% sequence identity in the region coding the VP1 capsid protein). However, three additional genetic clusters were identified from environmental surveillance isolates during January 2021–July 2022, again an indication of AFP surveillance gaps, although only one genetic cluster has been detected since June 2021.

Transmission of cVDPV2 from several emergences in Pakistan resulted in 165 cVDPV2 cases during July 2019–April 2021 (22 cases in 2019, 135 in 2020, and eight in 2021). In the most recent case, the patient had paralysis onset on April 23, 2021 (Table) (Figure 1) (Figure 2).

**TABLE:** Acute flaccid paralysis surveillance indicators, number of wild poliovirus cases reported, and number of circulating vaccine-derived poliovirus type 2 cases reported, by region and period — Pakistan, January 2021–July 2022

Region	AFP surveillance indicators				Poliomyelitis cases								
	No. of AFP cases (nonpolio AFP rate*)		% With adequate stool specimens^†^		Reported WPV1 cases					Reported cVDPV2 cases			
	2021	2022^§^	2021	2022	Jan–Jun 2021	Jul–Dec 2021	Jan–Jun 2022	Total		Jan–Jun 2021	Jul–Dec 2021	Jan–Jun 2022	Total
Azad Jammu Kashmir	273 (14.5)	212 (20.7)	89.7	93.4	0	0	0		**0**	0	0	0	**0**
Balochistan	564 (9.5)	245 (7.6)	87.4	89.4	1	0	0		**1**	4	0	0	**4**
Gilgit-Baltistan	129 (18.8)	88 (23.6)	83.0	84.1	0	0	0		**0**	0	0	0	**0**
Islamabad	172 (17.2)	104 (19.0)	84.3	81.7	0	0	0		**0**	0	0	0	**0**
Khyber Pakhtunkhwa	3,311 (15.8)	1,934 (17.5)	83.1	86.2	0	0	14		**14**	1	0	0	**1**
Punjab	6,300 (12.2)	3,705 (13.2)	84.2	87.9	0	0	0		**0**	1	0	0	**1**
Sindh	2,369 (10.5)	1,460 (11.9)	89.4	86.9	0	0	0		**0**	2	0	0	**2**
**Total**	**13,118 (13.0)**	**7,748 (14.2)**	**85.2**	**87.2**	**1**	**0**	**14**		**15**	**8**	**0**	**0**	**8**

**Abbreviations**: AFP = acute flaccid paralysis; cVDPV2 = circulating vaccine-derived poliovirus type 2; WPV1 = wild poliovirus type 1.

* Nonpolio AFP cases per 100,000 children and adolescents aged <15 years. Recommended benchmark: two cases per 100,000 persons aged <15 years.

^†^ Defined as two stool specimens collected ≥24 hours apart within 14 days of paralysis onset and arriving at a World Health Organization–accredited laboratory with reverse cold chain maintained and without leakage or desiccation.

^§^ Annualized.

## Discussion

The number of WPV1 cases and areas of poliovirus transmission identified in Pakistan declined markedly during January 2021–July 2022 compared with the preceding 2 years. The limited genetic divergence among WPV1 isolations since 2020 suggests that the reduction in cases and apparent geographic scope of virus spread are likely reflective of a decrease in WPV1 circulation during the reporting period. Disruptions to implementation of polio eradication activities because of the COVID-19 pandemic have been ameliorated since late 2020 (*1*,*6*), and a cVDPV2 outbreak that began in 2019 was interrupted by 2021 after a robust vaccination response.

The resurgence of WPV1 cases in 2022 and the isolation of WPV1 from environmental surveillance samples have demonstrated that Pakistan’s recent progress toward interrupting endemic WPV1 transmission has been jeopardized by persistent circulation in a historically challenging geographic area as well as by AFP surveillance limitations. Of note, an outbreak of WPV1 genetically linked to viral strains in Pakistan was identified during this period in Malawi and Mozambique, countries located in the WHO African Region, which was certified free of indigenous WPV1 transmission in September 2020 (*1*,*9*).

The clustering of WPV1 cases in North Waziristan demonstrates the progress of the Pakistan program in limiting the geographic scope of transmission. However, recent environmental surveillance isolations of WPV1 from sites in Islamabad and Punjab, genetically linked to WPV1 in circulation in southern Khyber Pakhtunkhwa, indicate the potential for further spread to other parts of the country. Months of flooding in the summer of 2022 in several areas of the country and associated displacement of persons, in addition to limiting the reach of SIAs, could increase the likelihood of further spread of the virus.

Although performance benchmarks for AFP surveillance are met nationally and at the provincial levels, priority should be given to enhancing the quality of AFP surveillance through continued training of health care workers and enlisting of more community informants. The planned expansion of environmental surveillance should be prioritized to include traditional poliovirus reservoirs without current environmental surveillance sites.

Despite high levels of poliovirus vaccination coverage during the reporting of SIAs nationally, the considerable challenges to quality of vaccination activities in the highest priority districts, as evidenced by repeatedly missed children and performance gaps indicated by LQAS survey results, could be addressed by improving microplanning of and supervision during SIAs in these areas. Postcampaign monitoring findings after the completion of vaccination activities should guide specific interventions focusing on areas with challenges to reaching and vaccinating children who are continually missed during SIAs. Vaccination activities should continue to be synchronized with neighboring Afghanistan, when and where feasible; tracking and vaccinating children in highly mobile populations and displaced families must also remain a priority for the Pakistan polio eradication program. With the return of targeted attacks on polio workers in Pakistan and Afghanistan by militants (*10*), efforts must be enhanced to ensure the safety of everyone serving at the frontlines of polio eradication.

There is an urgent need to take advantage of the window of opportunity presented by the current relative attenuation of poliovirus spread in Pakistan to end national and global transmission of WPV1. This will require expanding initiatives that foster more community engagement, providing incentives for participation in vaccination and empowering frontline polio workers, as well as mitigating the challenges posed by the flooding. To stop the spread of wild poliovirus in Pakistan and globally by the end of 2023, the country’s polio eradication program must ensure that no child is missed in the quest to administer life-saving vaccines.

**Summary**

**What is already known about this topic?**

Pakistan is one of two countries (including Afghanistan) where wild poliovirus type 1 (WPV1) transmission has never been interrupted.

**What is added by this report?**

WPV1 cases in Pakistan decreased from 147 in 2019 and 84 in 2020 to a single case in 2021 but increased to 14 cases in 2022 as of July 31. These 14 WPV1 cases are clustered among children in southern Khyber Pakhtunkhwa province, many of whom have never received poliovirus vaccine (zero-dose children).

**What are the implications for public health practice?**

Ensuring the highest quality vaccination activities in priority areas of Pakistan will enable the polio program to improve the chances of interrupting ongoing transmission of WPV1.

## Footnotes

* SIAs are mass house-to-house vaccination campaigns targeting children aged <5 years with OPV, irrespective of the child’s vaccination history.

†
https://www.gavi.org

§
https://polioeradication.org/news-post/global-eradication-of-wild-poliovirus-type-2-declared

¶ Based on priority history, current transmission patterns, and community polio vaccination status.

** LQAS surveys use a small sample size to assess the quality of vaccination activities within a few days after SIAs in union councils (i.e., subdistricts referred to as “lots”). LQAS surveys seek evidence of vaccination (i.e., finger marking) by random selection of 60 children within each lot. If the number of unvaccinated persons in the sample exceeds three, then the union council SIA is classified as having failed at a threshold of ≥90%, and additional vaccination activities in those areas are recommended. If the threshold of ≥90% (three or fewer unvaccinated children) is met, then the union council SIA is classified as having passed.

†† Nonpolio AFP cases are those that are discarded as not having laboratory or other proof of poliovirus as the cause. The expected background rate of nonpolio AFP illnesses is ≥2 per 100,000 children and adolescents aged <15 years per year, the standard WHO performance indicator target for sufficiently sensitive detection. The standard WHO stool specimen indicator target is adequate stool specimen collection from ≥80% of AFP cases.

§§ Stool specimens are considered adequate if two specimens are collected ≥24 hours apart within 14 days of paralysis onset and arrive at a WHO-accredited laboratory with reverse cold chain maintained and without leakage or desiccation.
